# The Critical Role of Transcription Factor RUNX2 in Bone Mechanobiology

**DOI:** 10.3390/cells15010050

**Published:** 2025-12-26

**Authors:** Maria A. Katsianou, Antonios N. Gargalionis, Kostas A. Papavassiliou, Angeliki Margoni, Athanasios G. Papavassiliou, Efthimia K. Basdra

**Affiliations:** 1 Department of Biological Chemistry, Medical School, National and Kapodistrian University of Athens, 11527 Athens, Greece; makatsianou@med.uoa.gr (M.A.K.); angeliki.margoni@gmail.com (A.M.); 2 Laboratory of Clinical Biochemistry, ‘Attikon’ University General Hospital, Medical School, National and Kapodistrian University of Athens, 12462 Athens, Greece; agargal@med.uoa.gr; 3 First University Department of Respiratory Medicine, ‘Sotiria’ Chest Hospital, Medical School, National and Kapodistrian University of Athens, 11527 Athens, Greece; konpapav@med.uoa.gr

**Keywords:** RUNX2, osteoblast, chondroblast, mechanical signals, mechanotransduction, bone remodeling

## Abstract

**Highlights:**

**What are the main findings?**

- Bone development and remodeling are strongly influenced by mechanical cues.
- RUNX2 acts as a pioneer transcription factor, integrating mechanical and molecular signals.

**What are the implications of the main findings?**

- Indirect regulation of RUNX2 via mTORC1 or microRNAs provides new therapeutic insights.
- Biomechanics-based therapies may help treat mechanically-driven bone disorders.

**Abstract:**

Mechanobiology plays a pivotal role in skeletal development and bone remodeling. Mechanical signals such as matrix stiffness, fluid shear stress, and hydrostatic pressure activate the Runt-related transcription factor 2 (RUNX2) bone-specific transcription factor through pathways including the mitogen-activated protein kinase (MAPK) signaling cascade and yes-associated protein (YAP)/transcriptional co-activator with PDZ-binding motif (TAZ) effectors. RUNX2 itself affects chromatin remodeling and nuclear architecture via Lamin A/C and Nesprin 1, thereby directing osteogenic differentiation. Thus, RUNX2 acts both as a mechanosensor and mechanoregulator, whereas RUNX2’s mechanosensitivity has been leveraged as a target to achieve bone regeneration. Notably, post-translational modifications and epigenetic alterations can orchestrate this regulation, integrating metabolic and circadian signals. However, due to RUNX2’s nuclear localization, its targeting remains a challenging issue. To this end, indirect targeting, through mammalian/mechanistic target of rapamycin complex 1 (mTORC1) or microRNAs (miRNAs), offers new strategies to employ biomechanics in an attempt to intervene with bone diseases driven by mechanical cues or degeneration, and ultimately repair and regenerate the damaged tissues. Herein we critically elaborate upon molecular aspects of RUNX2 regulation towards exploitation at the clinical level.

## 1. Introduction

The Runt-related transcription factor (RUNX) family, comprising Runt-related transcription factor 1 (RUNX1), Runt-related transcription factor 2 (RUNX2), and Runt-related transcription factor 3 (RUNX3) are vital for various physiological processes, such as cell lineage commitment, proliferation, and embryonic development. The common feature of the family members is the DNA-binding domain named ‘runt’ encompassing 128 amino acids [1]. RUNX1 is central for the maturation of the hematopoietic system and RUNX3 is essential for neuronal, gastrointestinal, and chondrocyte mutation, while RUNX2 is critical for osteogenic cell differentiation and bone formation [2]. Apart from bone remodeling, RUNX2 is implicated in pathophysiological processes, such as carcinogenesis and inflammation [3,4]. RUNX2 was firstly identified as a major transcription factor involved in osteogenesis and chondrocyte hypertrophy, directing the differentiation of mesenchymal stem cells (MSCs) into osteoblasts and regulating the expression of bone formation osteogenic genes, hence achieving bone stability [5,6,7,8]. Additionally, it is clearly documented that bone remodeling is prone to mechanical forces. Mechanical stimulation leads to augmentation of bone mass by modulating bone production and resorption, whilst the absence of such forces favors bone loss and diminishes tissue quality [9,10]. Bone is a dynamic tissue subjected to different mechanical stimuli, such as shear stress, compression, hydrostatic pressure, tension, and substrate stiffness. Matrix strain and fluid shear stress both cause cell deformation and trigger different signaling pathways [11]. Although greater responses have been seen under fluid flow, recent studies support that their effects on RUNX2 expression, whether they are direct or reproducible, remain variable and depend on experimental conditions, cell type, and the loading extent [12]. It is suggested that among these mechanical forces, tensile strain and substrate stiffness are upstream direct regulators of RUNX2 expression. More specifically, it was shown that the increased stiffness of a bone-microenvironment substrate promoted osteoblast adhesion, spreading, and RUNX2 upregulated expression, enhancing the osteoblast phenotype [13].

In vivo studies have shown that mechanical stretch can accelerate bone regeneration, and in vitro models conclude that stretching is conducive to maturation and differentiation of osteoblasts, as well as to bone matrix synthesis and mineralization [14,15]. Nevertheless, the underlying mechanisms through which mechanical stretch maintains osteoblast phenotype remain poorly understood.

Given the established role of mechanobiology in understanding how cells sense and respond to these mechanical cues, as well as the crucial role of RUNX2 in orchestrating bone differentiation, it is important to decipher the RUNX2-associated mechanotransduction processes [16]. Although RUNX2 is the master regulator of osteogenesis, recent data support its role as a mechanosensitive mediator of physical signals, that translates mechanical cues into specific transcriptional responses. A key question now arises: whether RUNX2 is merely a mechano-mediator or whether it modifies the mechanical microenvironment by itself. Herein, we propose a conceptual advance: that RUNX2 cannot only be viewed as a mechanosensitive transcription factor but also an active mediator of mechanical cues, a regulator of the mechanical microenvironment. RUNX2 interprets mechanical forces and strengthens the transcription of osteogenic genes, by modulating the microenvironment. This notion extends beyond existing evidence, which primarily describes RUNX2 as a passive mechano-transducer and instead declares RUNX2 as a central player in skeletal mechanobiology. In the present article we highlight this dual role of RUNX2, analyze its function in the alteration of nuclear architecture and skeletal mechanics, and explore its interplay with epigenetic mechanisms and signaling pathways, in order to elucidate current gaps and propose future prospects.

## 2. Extracellular Matrix and Cellular Responses

The extracellular matrix (ECM), being composed of collagens, glycoproteins, and proteoglycans, provides cells with structural support [17,18]. The ECM possesses two key mechanical properties, rigidity and stiffness; the former describes resistance to bending while the latter refers to resistance to elastic deformation. Cells’ capacity to sense and respond to mechanical cues from the ECM is vital for various biological processes; thus, ECM stiffness is critical. It is a mechanical property that mediates cell spreading, differentiation, migration, and proliferation, and, more importantly, defines stem cell differentiation and specificity [17,18]. Adherent cells assess the stiffness of their growth substrate by applying traction forces at attachment sites and evaluate how much the matrix was displaced in response to force. When the substrate is rigid, the limited displacement promotes the build-up of focal adhesions, F-actin assembly, and the establishment of intracellular tension. All these events lead to cell spreading. However, softer substrates do not provide internal tension, resulting in reduced spreading. It is worth mentioning that ECM stiffness is also dependent on the dimensions of the material, and the variability of loading magnitude levels or changes in material structure [19].

## 3. RUNX2 as a Modulator of the Mechanical Microenvironment

**Figure 1:**
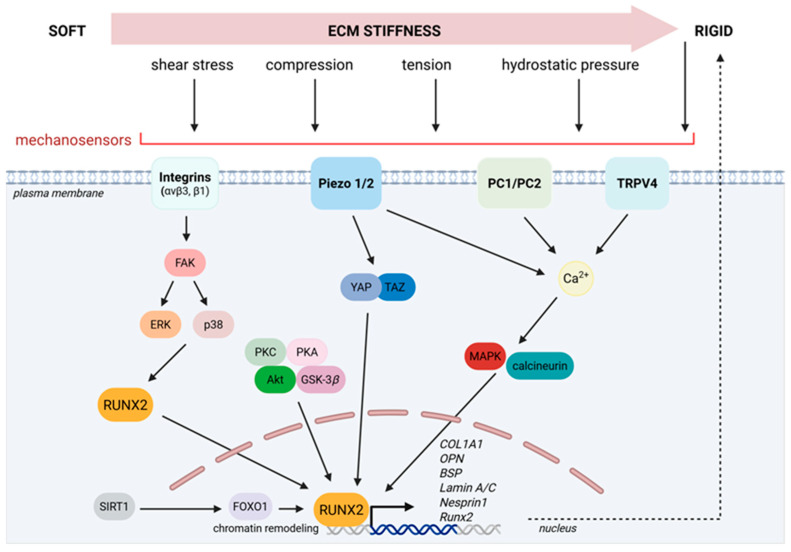
The mechanotransduction axis of RUNX2 in bone mechanobiology. Mechanosensors (integrins, ion channels, polycystins) sense mechanical stimuli (ECM stiffness, mechanical compression and tension, shear stress, hydrostatic pressure) and transmit the signals through a series of mechanotransducive kinases, transcription factors, and altered calcium influx, to regulate RUNX2 and osteogenic gene expression. The mechanisms involve nuclear mechanical re-organization, histone modifications, and chromatin remodeling as modulators of bone gene expression. Mechanotransducive gene transcription further enhances matrix rigidity. ECM, extracellular matrix; aνβ3–β1, integrin subunits; PC1/PC2, polycystins 1/2; TRPV4, transient receptor potential cation channel subfamily V member 4; FAK, focal adhesion kinase; ERK, extracellular signal-regulated kinases; RUNX2, Runt-related transcription factor 2; PKC, protein kinase C; PKA, protein kinase A; Akt, protein kinase B (PKB); GSK-3*β*, glycogen synthase kinase 3 beta; SIRT1, sirtuin 1; FOXO1, forkhead box protein O1; YAP, yes-associated protein; TAZ, transcriptional co-activator with PDZ-binding motif; MAPK, mitogen-activated protein kinase, COL1A1, collagen type 1 alpha 1; OPN, osteopontin; BSP, bone sialoprotein. Created in BioRender. Gargalionis, A. (2025) https://BioRender.com/24bcn2m.

ECM stiffness determines the differentiation of MSCs. Cells respond to stiffness via adhesion to matrix ligands [20]. A rigid bone microenvironment enhances osteogenesis and RUNX2 expression, while soft matrices enable the differentiation of various lineages [17]. ECM rigidity results in the spread of cells, fostering actomyosin contractility. This leads to increased focal adhesion formation, a step that affects downstream signaling. Such downstream signaling pathways engage transient receptor potential cation channel subfamily V member 4 (TRPV4), focal adhesion kinase (FAK), RhoA/Rho-associated protein kinase (ROCK), and downstream transcriptional regulators like Smad, *β*-catenin, and yes-associated protein (YAP) [21], which ultimately induce RUNX2 to promote osteoblast differentiation (Figure 1).

In other words, mediators of mechanical signals and ECM stiffness ‘collaborate’ with RUNX2, integrating extracellular stimuli to integrate the transcription of osteogenic genes and determine bone characteristics. Subsequently, RUNX2 responds to ECM stiffness by regulating ECM genes (*collagen type I alpha 1* (*COL1A1*)), *osteopontin* (*OPN*), *bone sialoprotein* (*BSP*)), altering the ECM mechanical properties [22].

ECM stiffness is linked to RUNX2 activation according to in vitro-matrix models. In these, MSCs are cultured on stiff hydrogels or substrates of varying elasticity. The substrate stiffness enhances focal adhesion formation, cytoskeletal contractility, and nuclear accumulation of RUNX2, accompanied by upregulation of osteogenic markers such as COL1A1, ALP, and OPN. MSC differentiation is further regulated through ERK or JNK activation [23,24]. However, in vivo mechanical-loading models, such as ulnar-loading models, support the idea that loading results in increased ERK/MAPK activity, RUNX2 phosphorylation, and osteoblast gene expression, independent of stiffness [25]. Presumably, these differences in dimensions (2D versus 3D), cell type, and presence of different cues can possibly influence RUNX2 presence and response, and thus should be considered when stiffness-driven mechanisms are incorporated in in vivo systems.

RUNX2 expression and activity is affected by the circadian rhythm. It interacts with core clock genes such as *Period 1* (*PER1*) and *Period 2* (*PER2*) [26,27]. Therefore, osteogenesis, being potentially controlled by the circadian rhythm, can be regarded as a metabolic–-mechanical process [27,28]. Based on recent data, RUNX2 and cellular metabolism interrelate under mechanical forces, ensuring that osteoblasts meet the energy requirements for matrix formation and remodeling [29]. Specifically, glucose metabolism is altered in mechanical stress via the sirtuin 1 (SIRT1)/forkhead box protein O1 (FOXO1) axis, affecting RUNX2 acetylation and stability [29].

Mechanical forces also induce the differentiation of MSCs, modulating the osteoblast cytoskeleton and nuclear architecture [30]. In vitro studies revealed the increased expression of osteogenic genes under fluid shear stress. Notably, the variations in magnitude, frequency, and volume of shear stress, in an oscillatory fluid flow model, were followed by changes in gene expression. A transient fluid flow stimulation induced the expression of *cyclooxygenase-2* (*COX2*), *OPN*, and *RUNX2* in the initial stages of osteogenesis, while prolonged stimulation increased the formation of collagen and matrix in the mature stage [31,32]. However, the absence of exact data on changes in RUNX2 activation in response to changes in shear magnitude does not allow reliable predictions of RUNX2 responses [32]. Notwithstanding the correlation of RUNX2 expression to stiffness and mechanical forces, the exact matrix mechanics that lead to RUNX2 activation need to be investigated through mechano-transcriptomics.

In order to achieve reproducible RUNX2 responses, a concrete experimental design should be followed, based on the combination of various shear stress levels, time-course lapses, and osteogenic cell models. To make this mechano-transcriptomic approach actionable, we propose an experimental plan in which MSCs, early osteoprogenitors, and mature osteoblasts are exposed to oscillatory or laminar fluid flow to physiologically relevant shear stresses (0.5–2.0 Pa) at various time frequencies (e.g., 15 min, 1 h, 4 h, and 24 h) [25]. Mechanical activation can be identified using time-resolved transcriptomic profiling (bulk or single-cell RNA sequencing) and ATAC-seq profiling to map shear dose- and time-dependent changes in transcription and chromatin architecture. Parallel kinase analyses (e.g., ERK, AKT) would also correlate RUNX2 to mechanosensitive pathways of upstream origin, followed by analyses of RUNX2 nuclear localization and post-translational regulation, allowing the identification of mechanical activation levels [33].

RUNX2 response to mechanical stimuli is defined not only by matrix rigidity, but also by viscoelasticity. Interestingly, three-dimensional hydrogels, mimicking bone composition, resulted in high levels of nuclear RUNX2 compared to two-dimensional substrates [34]. RUNX2 nuclear localization was assessed by confocal immunofluorescence microscopy, where the fluorescence intensity of the nuclear to cytoplasmic signal ratio of Runx2 (Runx2_nucleus_/Runx2_cytoplasm_) was quantified. RUNX2 activity was identified based on its nuclear translocation and on the use of transcriptional assays, focusing on downstream osteogenic target genes. Enhanced RUNX2 nuclear accumulation has been reported in various 3D material matrices including collagen, alginate, and supramolecular, across stiffness ranges related to bone marrow and pre-mineralized bone [35,36,37].

**Figure 2:**
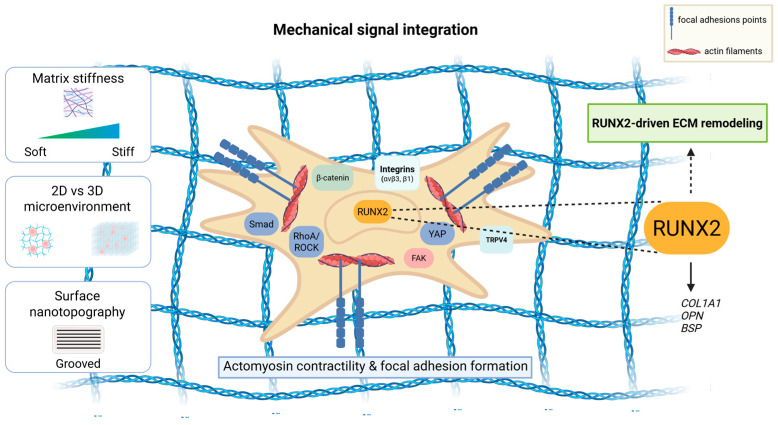
RUNX2 as a modulator of the mechanical microenvironment. Properties of the extracellular matrix, including matrix stiffness, dimensionality, and surface nanotopography, regulate cytoskeleton structure and focal adhesion points to activate mechanotransduction pathways mediated by RhoA/ROCK, Smad, integrins, β-catenin, FAK, YAP, and TRP channels. RUNX2 integrates these signals to regulate osteogenic gene expression. This further alters ECM mechanical properties and reinforces mechanosensitive signaling. BSP, bone sialoprotein; COL1A1, collagen type 1 alpha 1; ECM, extracellular matrix; FAK, focal adhesion kinase; OPN, osteopontin; RhoA, Ras homolog family member A; ROCK, Rho-associated coiled-coil-containing kinases; RUNX2, Runt-related transcription factor 2; TRPV4, transient receptor potential vanilloid 4; YAP, yes-associated protein. Created in BioRender. Gargalionis, A. (2025) https://BioRender.com/xdtqhbz.

RUNX2 signaling is also tuned by nanotopography, as surface topographical cues alter cytoskeletal stiffness and FAK—extracellular signal-regulated kinases (ERK) mitogen-activated protein kinase (MAPK) potentiation [38]. Further studies in bone marrow stromal cells correlate ECM glycation with reduced elasticity and subsequent RUNX2 mechanosensitivity [39] (Figure 2).

## 4. RUNX2 in Nuclear Mechanics

**Figure 3:**
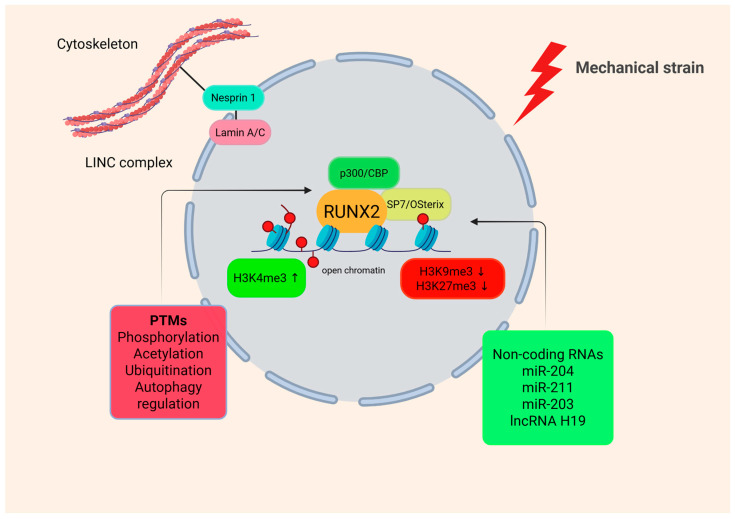
RUNX2 integrates mechanical and epigenetic signals within the nucleus. The cytoskeleton transmits mechanical signals through the LINC complex. This remodels nuclear architecture, permits RUNX2 to bind open chromatin, and recruits histone-modifying enzymes and transcriptional co-factors. Mechanical strain causes increase in activating histone modifications and suppression of repressive histone modifications. PTMs stabilize RUNX2 and mechanical stimulation suppresses inhibitory miRNAs but enhances lncRNA H19. These mechanisms support transcription of osteogenic genes. LINC, linker of nucleoskeleton and cytoskeleton; CBP, CREB-binding protein; RUNX2, Runt-related transcription factor 2; H3K4me3, histone H3 lysine 4 trimethylation; H3K9me3, histone H3 lysine 9 trimethylation; H3K27me3, histone H3 lysine 27 trimethylation; PTMs, post-translational modifications; lncRNA, long non-coding RNA. Created in BioRender. Gargalionis, A. (2025) https://BioRender.com/xj5sfce.

RUNX2, according to genetic and reprogramming analyses, is a pioneer transcription factor that contributes to the balance of chromatin accessibility, facilitating osteoblast differentiation through cell-type-specific gene expression [40]. It promotes the creation of accessible enhancer regions, via interaction with co-factors such as Sp7 (also called Osterix (Osx)) and histone-modifying enzymes, forming regulatory ‘hotspots’ (Figure 3).

Whether RUNX2 directly initiates chromatin opening or stabilizes accessibility in cooperation with co-factors, could be resolved by ATAC-seq following acute RUNX2 perturbation.

Mechanical signals alter RUNX2 binding accessibility, thus mechanical cues are linked to transcriptional regulation [41]. Nuclear membrane proteins Lamin A/C and Nesprin 1 expression are induced, with both being essential for nuclear remodeling and chromatin positioning (Figure 3). A deficient RUNX2 results in disruption of Lamin A/C binding to chromatin transcription start sites (TSS), thus impairing gene transcription and osteogenic differentiation [40]. The impaired chromatin organization that RUNX2 deficient-induced pluripotent stem cells (iPS) exhibit entails a malfunctional nuclear morphology, characterized by rigid nuclei or defects in chromosome segregation.

For osteoblast differentiation to occur, linkers such as RUNX2-induced linker of nucleoskeleton and cytoskeleton (LINC) complex regulate the establishment of the nuclear environment and membrane dynamics. Intracellular tension enables membrane stability through the actin skeleton, allowing Lamin A/C to bind to chromatin TSS regions, regulating gene expression and positioning chromatin to differentiation sites in a spatial manner. A dysfunctional RUNX2 has a profound impact on the nuclear environment and membrane. It affects its morphology and rigidity, modulating the ability to respond to stimuli, as well as osteoblast differentiation induced by RUNX2 [42]. Defective or complete loss of RUNX2 function results in a changed nuclear morphology, less rigid nuclei, and decreased levels of intracellular mechanical tension in human cells. Restoring Nesprin expression can rescue the defects of nuclear stiffness and intracellular tension in RUNX2-deficient cells, underscoring RUNX2’s role supporting the linkage between the cytoskeleton and the nucleus [40]. We hypothesize that for an optimal RUNX2-mediated gene expression to be achieved, it may require nuclear mechano-regulation, suggesting a unique RUNX2 function that could transcend classical transcriptional regulation.

Although these findings indicate that RUNX2 acts upstream of the LINC complex by inducing Nesprin-expression, the direct binding of RUNX2 to regulatory element spectrin repeat-containing nuclear envelope protein (SYNE1), was not directly elucidated. Future experiments, such as RUNX2 Chi-seq at the SYNE1 locus and RUNX2 suppression followed by analysis of newly synthesized RNA would be required to definitively distinguish direct from indirect transcriptional regulation. These data highlight RUNX2 as a central component of mechanotransduction-chromatin remodeling, integrating signals elicited by ECM stiffness, through mechanosensory molecules and via signaling pathways and nuclear dynamics, which all impact on epigenetic mechanisms to control an osteogenic status. Notably, answers to questions on how RUNX2 independently invades condensed chromatin to initiate this regulatory action and its interaction with the above key nuclear proteins, as well as the effect on enhancer accessibility and how mechanical forces relate to its multifarious function remain elusive.

## 5. Ion Channels and RUNX2 Interplay

**Figure 4:**
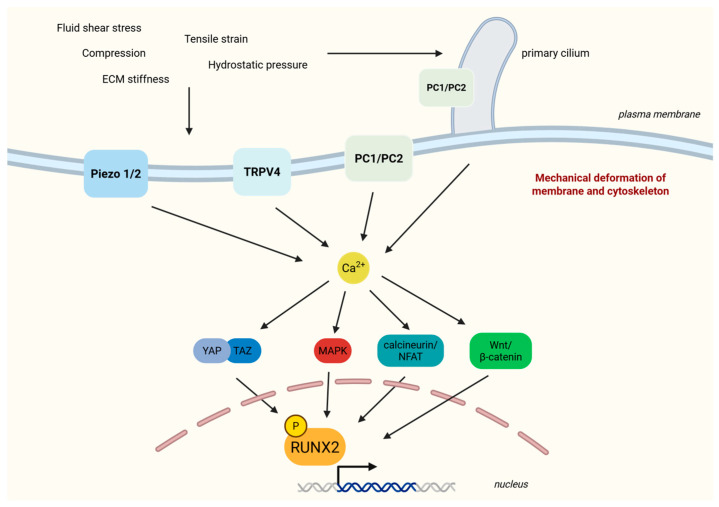
Ion channels and RUNX2 interplay in bone mechanotransduction. Mechanical stimuli (fluid shear stress, tensile strain, compression, hydrostatic pressure, ECM stiffness) cause mechanical deformation of membrane and cytoskeleton. Piezo 1/2 are activated, TRPV4 senses osmotic and mechanical strain, polycystins form mechano-induced ion channels at the cell membrane and at the primary cilia, and cilia bend under fluid flow. Ion channels permit calcium transients and signaling through YAP/TAZ, MAPK, calcineurin/NFAT, and Wnt/β-catenin pathways. Ca^2+^-dependent kinase activation promotes RUNX2 phosphorylation and nuclear accumulation. ECM, extracellular matrix; MAPK, mitogen-activated protein kinase; NFAT, nuclear factor of activated T-cells; PC1/PC2, polycystins 1/2; RUNX2, Runt-related transcription factor 2; TAZ, transcriptional co-activator with PDZ-binding motif; TRPV4, transient receptor potential cation channel subfamily V member 4; YAP, yes-associated protein. Created in BioRender. Gargalionis, A. (2025) https://BioRender.com/pnnozl1.

Cells employ mechanosensitive molecules and ion channels to detect stimulation by ECM stiffness. This requires regulators such as RUNX2. Mechanical stimulation takes place through a complex interconnection between mechanosensory molecules such as Piezo 1/2 ion channels, integrins, and primary cilia, which trigger intracellular signaling pathways (MAPK, Wingless-related integration site (Wnt)/*β*-catenin, YAP/TAZ) [3] (Figure 4).

Through *a*ν*β*3 and *β*1 subunits, integrins manage to translate mechanical forces such as matrix stiffness, tensile strain, or compression into mechanical cues that activate RUNX2. FAK phosphorylation and potentiation of the downstream effectors MAPK ERK and p38 enhance RUNX2 activity [30]. Additional sensors, including polycystins and the LINC complex, bridge cytoskeleton strain to nuclear formation/deformation to ultimately regulate RUNX2 [43]. RUNX2 cooperates with mechanosensitive complexes like polycystins to integrate extracellular matrix rigidity cues—a vital signaling integration during both embryonic skeletal development and adult bone remodeling, as deficient mechanotransduction through RUNX2 results in impaired osteogenesis and skeletal abnormalities (Figure 4) [3].

Chromatin organization and RUNX2 accessibility to target promoters is influenced by mechanical loading [40]. Piezo 1 is a major skeletal mechanosensor that, in response to mechanical forces, controls YAP/TAZ-dependent collagen expression and bone homeostasis [44]. Piezo 1/2 and transient receptor potential (TRP) channels identify membrane stretching and fluid flow, generating calcium transients that activate downstream signaling cascades, including MAPK and calcineurin pathways, altering the nuclear localization and phosphorylation of RUNX2 (Figure 4) [45].

However, data demonstrate that transient activation of Piezo alone does not suffice for osteogenic transcription; sustaining RUNX2 signaling requires cytoskeleton interaction transmitted through the integrin—FAK axis, while YAP/TAZ and calcineurin–NFAT pathways further reinforce osteogenic gene expression programs [46]. While Piezo activation and mechanical cues result in dose-dependent calcium signaling and mechano-activation, including YAP/TAZ and calcineurin-NFAT signaling, dose response relating to sustained RUNX2-dependent transcription has not yet been established [21,45]. Future studies should examine the mechanical dose and duration required for optimal RUNX2 activation. Specifically, the questions arising are whether RUNX2 responds differently to transient and chronic strain and if RUNX2 could adjust its activity based on the strength of the stimulus or time lapses.

During osteogenic differentiation, multiple kinases, including MAPK ERK, protein kinase C (PKC), protein kinase A (PKA), protein kinase B (PKB; also termed AKT), cyclin-dependent kinase 2 (CDK2), glycogen synthase kinase 3 beta (GSK-3*β*), and cyclin-dependent kinase 4 (CDK4) target distinct serine/threonine residues on RUNX2, resulting in either potentiation or inhibition of its transcriptional activity. Mechanical stimulation activates signaling pathways such as ERK and AKT, which are shown to phosphorylate RUNX2 at sites associated with increased stability and osteogenic gene expression [45]. However, phosphorylation mediated by GSK-3β or cyclin-dependent kinases has been linked to reduced RUNX2 activity or enhanced degradation [47]. In order to dissect how competing phosphorylation events on RUNX2 are hierarchically integrated under different mechanical conditions, such as cyclic strain versus static loading, further experiments would be vital—for example, an applied matched cyclic strain and static loading at different time points, along with a RUNX2 dominance quantification on phosphosite sites, using functional assessment such as CRISPRi. Early studies identified RUNX2 phosphorylation induced by ERK at Ser302, a key stabilization event [48]. Phospho-proteomic evidence reconfirms that RUNX2 is positioned at the crossroad of multiple mechanoresponsive kinases, including ERK, AKT, and GSK-3*β* [49].

## 6. Epigenetic and Post-Translational Modifications

**Figure 5:**
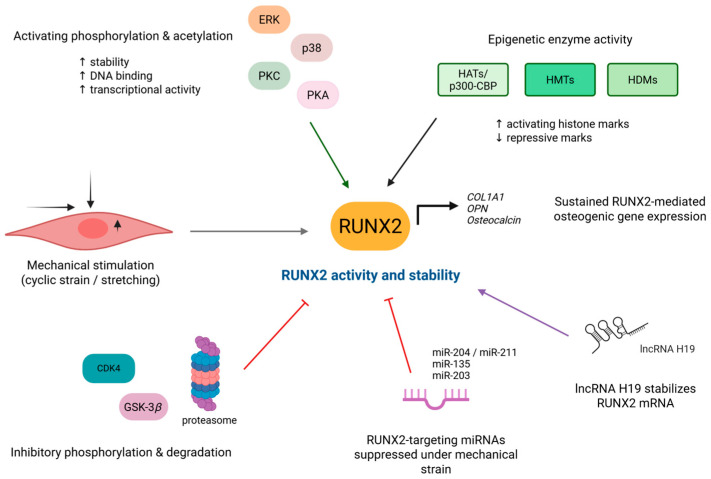
Post-translational and epigenetic modulation of RUNX2 under mechanical stimulation. RUNX2 functions as a molecular hub to regulate its activity and stability. Under mechanical stimuli, such as cyclic strain or stretching, RUNX2 is activated through post-translational modifications, but also inhibited through phosphorylation that leads to proteasomal degradation. Mechanical cues favor permissive epigenetic enzyme activity through histone acetyltransferases, histone methyltransferases, and histone demethylases to stabilize RUNX2. Mechanical strain suppresses RUNX2-targeting miRNAs, while lncRNA H19 stabilizes RUNX2mRNA. These events lead to sustained RUNX2-mediated osteogenic gene expression. CBP, CREB-binding protein; CDK4, cyclin-dependent kinase 4; COL1A1, collagen type 1 alpha 1; ERK, extracellular signal-regulated kinases; GSK-3β, glycogen synthase kinase 3 beta; HAT, histone acetyltransferase; HDM, histone demethylase; HMT, histone methyltransferase; OPN, osteopontin; PKA, protein kinase A; PKC, protein kinase C; RUNX2, Runt-related transcription factor 2. Created in BioRender. Gargalionis, A. (2025) https://BioRender.com/27oty4h.

Epigenetic alterations such as DNA methylation, histone acetylation, and methylation modulate chromatin structure close to RUNX2-regulated loci, coordinating osteoblast differentiation (Figure 5).

Histone marks either increase or decrease during mechanical stimulation. Histone H3 lysine 4 trimethylation (H3K4me3), due to osteogenic activation, increases at RUNX2 promoters, while histone H3 lysine 9 trimethylation (H3K9me3) and histone H3 lysine 27 trimethylation (H3K27me3) are suppressive and marks a decrease leading to an open chromatin structure which contributes to *RUNX2* expression [50]. H3K9 trimethylation is catalyzed by the histone methyltransferase SETDB1, which inhibits gene transcription through heterochromatin formation. Dysfunctional SETDB1 activity has been linked to osteosarcoma and other malignancies, highlighting the importance of a properly regulated H3K9 methylation in bone-associated genes [51]. Chromatin changes were mapped specifically at the promoters of RUNX2 and OSX using ChIP-qPCR [50]. However, these experiments were performed under osteo-inductive conditions rather than defined mechanical stimulation conditions. Future studies combining ChIP-seq or CUT&RUN combined with defined loading protocols will be required to establish a direct causality between mechanical forces, RUNX2 occupancy, and histone modification dynamics.

In addition to epigenetic modifications, RUNX2 is prone to post-translational modifications (PTMs), including phosphorylation, acetylation, and ubiquitination, all controlling its activity [52]. Also, non-coding RNAs influence RUNX2 mechanoregulation. MicroRNAs (miRNAs) that typically target RUNX2 are suppressed under mechanical stretch, yet long non-coding RNA H19 (lncRNA H19) increases, acting as an osteogenesis trigger and stabilizer of RUNX2 mRNA [53]. Although these RNA modulators control RUNX2 stability and its transcriptional capacity under various mechanical inputs, their functional roles in vivo under varied mechanical loading remain unresolved. Specific gain- or loss-of function approaches should be employed combined with mechanical unloading, overload, or microgravity models to directly assess the mechanical control of RUNX2.

In pre-osteoblastic cells and osteoblasts, RUNX2 phosphorylation at serine and threonine residues within its proline/serine-rich domain is ERK- and p38-dependent. Under mechanical stimuli, such as cyclic strain or stretching, RUNX2 accumulates in the nucleus leading to transcription of osteogenic target genes such as *COL1A1*, *OPN*, and *osteocalcin* [54]. ERK-guided phosphorylation of RUNX2, owing to mechanical forces, augments its DNA-binding potential and subsequent activation of bone matrix genes. The stabilization of RUNX2 is further accomplished by acetylation, preventing proteolysis, thereby maintaining mechanotransduction [55]. Autophagic pathways, which connect cellular responses to transcriptional regulation, also affect RUNX2 stability. Mice under mechanical strain, lacking autophagy-related genes, displayed elevated levels of RUNX2 phosphorylation, while basal expression was decreased [56]. The co-existence of mechanical stimuli along with suppressed autophagy has cumulative effects on RUNX2 downregulation, reducing bone formation, ATP release, and ERK signaling [56].

The abundance of RUNX2 is also modulated by mammalian/mechanistic target of rapamycin (mTOR) and nuclear factor kappa B (NF-κB) in osteoblast-like cells, which upregulate RUNX2 in response to mechanical forces. The associated inhibition of both factors under strain maintains RUNX2 activity and osteoblast homeostasis [57]. Consequently, RUNX2’s ‘duality’, both as a target and an enhancer of mechanical signals, assists in controlling bone homeostasis and related disorders.

Other activation post-translational modifications, such as phosphorylation by protein kinase C delta (PKCδ) at Ser247 and by protein kinase A (PKA) at Ser28, Thr340, and Ser347 act in parallel and similarly increase RUNX2 expression [58,59]. The recruitment of co-activators such as p300/CREB-binding protein (CBP) and histone acetyltransferases (HATs) is enabled by these phosphorylation events. In contrast, GSK-3*β*-mediated phosphorylation at Ser369-Ser377 or cyclin D1/CDK4 at Ser472 suppress RUNX2 through proteostasis [47]. Evidently, RUNX2 acts as a mechano-effector in the nucleus, translating stimuli into transcriptional events that regulate osteogenesis and bone formation, hence linking mechanotransduction to the nucleus [16]. Consequently, RUNX2 PTMs and epigenetic regulation offer promising therapeutic targets for combating diseases influenced by mechanical forces.

## 7. Clinical Perspectives

At the clinical level, RUNX2 is a central transcription factor involved in bone diseases and cancers. RUNX2 haploinsufficiency causes cleidocranial dysplasia (CCD), a rare genetic disorder associated with skeletal dysfunction and dental abnormalities, whereas its continuous activation can result in craniosynostosis, the premature closure of cranial sutures [60,61]. Apart from bone biology, RUNX2 is implicated in osteoarthritis (OA), where its overexpression is linked to OA emergence. Increased expression of RUNX2 in human and animal OA cartilage is related to the expression of matrix-degrading enzymes of the articular chondrocytes [62,63]. Therefore, RUNX2 is involved in OA pathogenesis, highlighting its role as a potential therapeutic target. It is suggested that miRNAs could possibly target RUNX2, since in OA patients, in both serum and cartilage tissue, miRNA expression was altered [62]. In osteoporosis, meta-analysis research proved that *RUNX2* T > C polymorphisms can affect bone homeostasis in women after menopause and can effectively predict the disease. Specifically, women with CC genotypes exhibited a lower spine bone mineral density (BMD) in comparison to women with wild-type genotypes [64].

RUNX2 and its multifaceted role is not only restricted in bone disease but also in oncogenesis and metastasis. A recent study revealed that RUNX2 affects cancer metabolism inducing plasticity and metastasis. The tumour microenvironment, such as matrix stiffness and composition, activate mechanotransduction pathways (e.g., integrin–FAK, YAP/TAZ) that promote EMT and metabolic reprogramming, processes intersecting with RUNX2–sterol regulatory element-binding protein 1 (SREBP1) signaling. SREBP1 activation has been reported in stiff tumor environments suggesting that biomechanical forces can trigger the RUNX2–SREBP1 relationship. This rationale can be tested using 3D tumor spheroids or organoids with tunable stiffness, coupled with RUNX2–SREBP1 inhibition and metabolic assays.

Embryonic transcription factors that are re-activated in cancer, and RUNX2, despite being a typical regulator of osteogenesis, promotes metastasis through trans-differentiation processes like epithelial-to-mesenchymal transition (EMT) and osteomimicry [65]. RUNX2, by co-operating with SREBP1, accomplishes the upregulation of genes involved in lipid biosynthesis [66]. An important finding in thyroid and breast cancer samples is that the metastatic potential is correlated with co-expression of RUNX2 and SREBP1 [65]. Therefore, RUNX2 is involved in both translational and metabolic adaptation. Studies have implicated RUNX2 in migration of solid tumor cells where RUNX2 overexpression induces cancer cell invasion and metastasis [67,68,69]. In triple-negative breast cancer, RUNX2 silencing suppressed cancer cell proliferation, metastasis, and invasion as well as chemoresistance. Notably, the RUNX2 and matrix metalloproteinase 1 (MMP1) axis serves as a new marker for breast cancer diagnosis [68].

Aberrant expression of RUNX2 is also an osteosarcoma feature, along with a loss in p53 and miR-34 expression. A highly expressed RUNX2 may activate, at a transcriptional level, tumor-promoting genes such as *OPN*, whereas RUNX2 depletion can hamper lung metastasis in vivo [70]. RUNX2, based on bioinformatics data, can act as a novel prognostic biomarker and a promising target in lung and pancreatic cancer [71]. In colon cancer cells, invasion and migration levels are positively regulated by RUNX2 and Brahma-related gene 1 (BRG1) which form a complex regulating cluster of differentiation 44 (CD44) signaling pathway [72]. Future studies should aim to experiment on tumors that express high RUNX2 levels to test bone microenvironment therapies, or emerging RUNX2 inhibitors.

In addition to its role in tumor biology, RUNX2 is implicated in the vasculature. In the context of aortic valve stenosis (the most common calcific aortic valve disease (CAVD) in high-income countries), RUNX2 is expressed early and is essential for the osteochondrogenic differentiation of aortic cells [73]. High levels of RUNX2 in vascular smooth muscle cells can lead to osteogenic reprogramming causing calcification of the valves and aortic valve stenosis [73,74]. In the clinical setting, specifically in ageing and metabolic diseases where one of the hallmarks is vascular mineralization, RUNX2 could be a valuable therapeutic target [52].

In skeletal disorders, RUNX2 is strongly influenced by mechanical loading, whereas in cancer, vascular mineralization, and inflammatory cartilage degeneration, RUNX2 dysregulation is more dependent on metabolic, inflammatory, or developmental cues. Distinguishing between regulatory contexts is essential when targeting RUNX2. Therapies aimed at mechanotransduction are likely to be most effective in strain-sensitive tissues, whereas specific approaches may be required in non-mechanical disease settings.

Indirect modulation of RUNX2 via upstream signaling pathways (e.g., mTORC1) or RNA-based regimens represent promising therapeutic strategies. It has been demonstrated that sirtuin 6 (SIRT6) deacetylates RUNX2 leading to its ubiquitination, inhibiting vascular smooth muscle osteogenic differentiation and subsequent calcification [75]. Experimental data also highlighted the contribution of mTOR/regulatory-associated protein of mTOR (Raptor) to osteoblast biology, implying a genetic interaction between Raptor and RUNX2. It was shown that mTOR/Raptor signaling is essential for bone formation in vivo through regulation of RUNX2 expression [76].

An indirect modulation of RUNX2 via upstream pathways can alter mechanobiology-relevant outcomes. More specifically, a preclinical model modulating RUNX2 indirectly via exosomes showed that in both β-glycerophosphate-induced VSMC calcification in vitro and vitamin D3-induced vascular calcification in vivo, BMSC-derived exosomes markedly reduced VSMC calcification [77]. However, key experimental gaps remain, including the confirmation that the effects are RUNX2-dependent, the evaluation of different vascular properties, and the determination of whether through cell-specific-delivery long-term efficacy is achieved. Interestingly, several studies claim that specific miRNAs (e.g., miR-204/211, miR-135, miR-203), by downregulating RUNX2, can inhibit cellular processes such as osteogenesis and metastasis [78,79].

Collectively, RUNX2 is activated across different tissues such as bone, cartilage, vasculature, and cancer. Consequently, it is a transcription factor integrating several osteogenic, metabolic, and oncogenic signalling pathways. Understanding how external cues, such as mechanical forces, mechanical stress, or inflammation, control RUNX2 expression could set the precedent of identifying new targets and treatment approaches.

## 8. Conclusions

RUNX2 has a dual role in mechanobiology, acting as both a mechanotransducer and mechano-effector, sensing and translating mechanical cues and integrating mechanical, biochemical, and epigenetic signals to orchestrate osteoblast differentiation by remodeling nuclear architecture and extracellular matrix rigidity. RUNX2 localization and transcriptional potential are altered through key pathways (FAK/ERK, YAP/TAZ), and RUNX2 itself regulates several genes (*COL1A1*, *OPN*, *BSP*), linking mechanical signals to osteoblast or oncogenic gene expression. Moreover, through post-translational and epigenetic modifications, RUNX2 integrates mechanical, metabolic, and circadian signals to balance osteoblast differentiation and bone remodeling.

Dysregulated RUNX2 signaling contributes not only to pathologies in skeletogenesis such as cleidocranial dysplasia, osteoporosis, and OA, but also vascular calcification and cancer progression and metastasis. Thus, its multifactorial role provides a vast array of potential targets that are being preclinically and clinically evaluated. What remains an open question is the interplay between RUNX2 and other nuclear factors such as YAP/TAZ, and how these factors contribute to its role in nuclear architecture and subsequent gene accessibility.

Future work should focus on simulating more accurate cell microenvironments to decode the way mechanical dose, timing, and chromatin context shape RUNX2 signaling. Advances in transcriptomics and epigenomics will enable mapping of RUNX2 networks under varying cues, illuminating how chromatin organization responds to mechanical loads. The implementation of bioengineering approaches such as biomaterials, could further harness RUNX2 potential to act as a mechanosensor. More specifically, a future experimental roadmap should integrate tunable hydrogels to control mechanical dose and timing with live-cell imaging of RUNX2 dynamics, such as nuclear localization under specific mechanical cues. Multi-omics should also be utilized, including transcriptomics, chromatin accessibility, and phosphoproteomics, using RUNX2 gain- or loss-of-function experiments and validated in bone, tumor, and vascular models.

Undoubtedly, RUNX2 is a dynamic translator of various kinds of mechanical cues and a controller of mechanically-triggered genome reprograming. Further insights into its role will contribute to more precise therapeutic strategies for skeletal, malignant, and vascular diseases.

## Data Availability

No new data were created or analyzed in this study.

## Abbreviations

The following abbreviations are used in this manuscript:

AKT	Protein kinase B (PKB)
aνβ3–β1	Integrin subunits
BMD	Bone mineral density
BMSC	Bone marrow mesenchymal stem cell
BRG1	Brahma-related gene 1
BSP	Bone sialoprotein
CAVD	Calcific aortic valve disease
CBP	CREB-binding protein
ChIP	Chromatin immunoprecipitation
ChIP-qPCR	Chromatin immunoprecipitation followed by quantitative polymerase chain reaction
CD44	Cluster of differentiation 44
CDK2	Cyclin-dependent kinase 2
CDK4	Cyclin-dependent kinase 4
CCD	Cleidocranial dysplasia
COL1A1	Collagen type 1 alpha 1
COX2	Cyclooxygenase-2
ECM	Extracellular matrix
EMT	Epithelial-to-mesenchymal transition
ERK	Extracellular signal-regulated kinases
FAK	Focal adhesion kinase
FOXO1	Forkhead box protein O1
GSK-3*β*	Glycogen synthase kinase 3 beta
HAT	Histone demethylase
HDM	Histone acetyltransferase
H3K4me3	Histone h3 lysine 4 trimethylation
H3K9me3	Histone h3 lysine 9 trimethylation
H3K27me3	Histone h3 lysine 27 trimethylation
iPS cells	Induced pluripotent stem cells
lncRNA	Long non-coding RNA
lncRNA H19	Long non-coding RNA H19
LINC	Linker of nucleoskeleton and cytoskeleton
MAPK	Mitogen-activated protein kinase
miRNA	MicroRNA
mTOR	Mammalian/mechanistic target of rapamycin
mTORC1	Mammalian/mechanistic target of rapamycin complex 1
MSCs	Mesenchymal stem cells
MMP1	Matrix metalloproteinase 1
NFAT	Nuclear factor of activated T-cells
NF-κB	Nuclear factor kappa B
OA	Osteoarthritis
OPN	Osteopontin
PC1/PC2	Polycystins 1/2
PKA	Protein kinase A
PKC/PKCδ	Protein kinase c/protein kinase c delta
PTMs	Post-translational modifications
RNA	Ribonucleic acid
RhoA	Ras homolog family number A
ROCK	Rho-associated coiled-coil-containing kinases
RUNX	Runt-related transcription factor
RUNX1	Runt-related transcription factor 1
RUNX2	Runt-related transcription factor 2
RUNX3	Runt-related transcription factor 3
SETDB1	Histone-lysine N-methyltransferase SETDB1
SIRT1	Sirtuin 1
SIRT6	Sirtuin 6
Smad	Suppressor of mothers against decapentaplegic
Sp7	Transcription factor Sp7
SREBP1	Sterol regulatory element-binding protein 1
SYNE1	Spectrin repeat-containing nuclear envelope protein
TSS	Transcription start site
TAZ	Transcriptional co-activator with PDZ-binding motif
TRP	Transient receptor potential
TRPV4	Transient receptor potential cation channel subfamily V member 4
Wnt	Wingless-related integration site
YAP	Yes-associated protein
